# The Use of Filgrastim in Patients with Hodgkin Lymphoma Receiving ABVD

**Published:** 2017-10-01

**Authors:** Adam F. Binder, Sonia Rai, Amir Steinberg

**Affiliations:** 1 Montefiore Medical Center, 111 East 210th Street Bronx, New York, NY, 10467, USA; 2 Icahn School of Medicine at Mount Sinai 1 Gustave L. Levy Place New York, New York, NY, 10029, USA

**Keywords:** Bleomycin, Granulocyte stimulating factor, Bleomycin induced-pulmonary injury, Hodgkin lymphoma, Treatment complications

## Abstract

**Background:** There is conflicting data about the increased risk of pulmonary toxicity when granulocyte-stimulating factor (G-CSF) is given in combination with bleomycin. No clear consensus for management of patients with Hodgkin lymphoma (HL) who require G-CSF support exists. Our objective was to evaluate whether there is an increase in pulmonary toxicity in patients who receive bleomycin and G-CSF during treatment for HL.

**Materials and Methods:** We conducted a single-center retrospective analysis of patients with Hodgkin Lymphoma from January 2003 until July 2015. All patients who received at least 1 dose of bleomycin and followed at our institution were included. Patients were evaluated for pulmonary toxicity starting from the day of first dose of bleomycin until 1 year after initiation of bleomycin. Data on pre-identified risk factors for pulmonary toxicity were also collected.

**Results:** Fifty-four patients met inclusion criteria. Twenty-one patients received bleomycin alone, and 33 patients received bleomycin and G-CSF. There was no statistically significant (p = 0.50) difference in the development of pulmonary toxicity between the two groups. Crude hazard ratio for development of pulmonary toxicity in the bleomycin and G-CSF cohort was 1.58 (95% confidence interval, CI: 0.41-6.12). On multivariate analysis, the hazard ratio for development of pulmonary toxicity was 1.71 (95% CI: 0.43-6.81).

**Conclusion**: This study does not find evidence that the combination of bleomycin and G-CSF increases the risk for bleomycin- induced pulmonary toxicity. We recommend G-CSF use in HL patients receiving bleomycin when needed to maintain dose intensity.

## Introduction

 ABVD (Adriamycin, Bleomycin, Vinblastine, Dacarbazine) is the standard regimen used for most Hodgkin lymphoma (HL) patients. It was initially developed in 1973 by “the Milan Group” (Milan Cancer Institute)^   1 ^. In the early 1990s, ABVD became the standard of care for Hodgkin lymphoma. In a pivotal trial by Canellos et al., ABVD was noted to have better failure-free survival with less toxicity compared to MOPP (Nitrogen Mustard, Vincristine, Procarbazine, Prednisone) chemotherapy^2^. In this seminal trial, 6% of patients receiving ABVD experienced bleomycin-induced pulmonary toxicity (BIP). The FDA did not approve Filgrastim (G-CSF) until February 20^th^, 1991, and thus it was not used for patients in the Canellos study^2^^,^^3^. In a rat model of bleomycin-induced lung toxicity, G-CSF increased alveolar neutrophil recruitment, pulmonary edema, and lung myeloperoxidase activity^        4 ^. Subsequently, concerns have been raised as to whether the use of G-CSF further increases pulmonary toxicity when used in combination with bleomycin-containing regimens. In humans, however, there has been conflicting data on the use of growth factor support in HL patients and the development of bleomycin- induced pulmonary toxicity.

In one retrospective study of Hodgkin lymphoma patients receiving ABVD, the investigators concluded that patients were at an increased risk for BIP if they were over the age of 40 or received G-CSF^        5 ^. Another study, the largest to date, revealed that low albumin and G-CSF were risk factors for BIP, but age, smoking history and pre-existing lung function were not risk factors^   6 ^. Contradictory to this, in other studies, there did not appear to be an association of G-CSF use with increased risk for BIP^7^^,^^8^. In addition, a smaller study by Younes et al.^        9 ^ evaluating the use of pegfilgrastim in patients receiving ABVD had a rate of pulmonary toxicity within an acceptable range and was similar to studies of patients receiving bleomycin alone.

BEACOPP (Bleomycin, Etoposide, Adriamycin, Cyclophosphamide, Vincristine, Procarbazine, Prednisone), a more intense regimen developed by the German Hodgkin’s Lymphoma Study Group, routinely incorporates growth factor support such as G-CSF into its regimen^10^^,^^11^. In one of these studies, grade 3-4 respiratory tract adverse effects occurred in 4-6% of the patient population. When comparing standard dose BEACOPP without growth factor support to increased dose BEACOPP with G-CSF, no difference in ‘respiratory tract effects’ was demonstrated, but actual rates of BIP vs. infection-related complications were not reported. 

Currently, the National Comprehensive Cancer Network (NCCN) does not recommend routine growth factor support during ABVD therapy. G-CSF is not recommended because ABVD is considered intermediate-risk for neutropenic fever^12^^,^^13^. There are examples in the literature supporting this approach; Boleti and Mead published a retrospective study of 38 Hodgkin lymphoma patients, 95% of whom completed their therapies without dose adjustments and none of the patients used growth factor support^        14 ^. Evans et al. retrospectively analyzed 59 HL patients, and noted minimal interruption to ABVD use in this patient population who did not receive G-CSF^        15 ^.

In the Phase III Millennium Pharmaceuticals-Seattle Genetics joint study comparing ABVD to ABV+brentuximab vedotin in stage 3 and 4 Hodgkin lymphoma patients, growth factor use was left to the discretion of the site investigators.^   16 ^ In discussion with site investigators as well as experts outside the context of this study, there is no uniform practice pattern regarding the use of growth factor support with ABVD combination chemotherapy for HL patients. 

Given this conflicting data, we set out to develop institutional guidelines for the use of G-CSF when administering combination chemotherapy that includes bleomycin. In developing these guidelines, we designed a single center retrospective study to evaluate whether there was evidence for an increase in bleomycin-induced pulmonary toxicity in patients who received G-CSF. 

## MATERIALS AND METHODS

 We conducted a retrospective analysis of patients diagnosed with Hodgkin lymphoma at our institution from January, 2003 until July, 2015. The Mount Sinai Data Warehouse was used to collect all available data. A search was conducted for all patients who had received at least 1 dose of bleomycin during the specified time period. Individuals, who did not carry a diagnosis of Hodgkin lymphoma, were excluded. Furthermore, those patients diagnosed at our center but received treatment elsewhere were excluded from the study. The 54 remaining patients were available for analysis. 

Patients who had received bleomycin for treatment of their HL were stratified by whether or not they had received G-CSF during the treatment period. Three reviewers (AB, SR, AS) performed chart reviews to identify evidence of pulmonary toxicity during the period starting from the day of the first dose of bleomycin until 1 year after the first dose of bleomycin. Pulmonary toxicity was defined as documentation of respiratory symptoms and one of the following: chest radiograph infiltrate with negative infectious workup, CAT scan pulmonary infiltrate with negative infectious workup, decline in DLCO, or bleomycin discontinuation due to respiratory symptoms without other findings (Table 1). 

**Table 1:** Definition of Pulmonary Toxicity

**Pulmonary toxicity was defined as respiratory symptoms and one of** **the following:**
Chest X-ray with pulmonary infiltrate and negative infectious work up
CAT scan with pulmonary infiltrate and negative infectious work up
Decline in DLCO
Bleomycin discontinuation due to respiratory symptoms without other findings

Data on pre-identified risk factors for pulmonary toxicity were also collected^5^^,^^17^^-^^19^. Risk factors for pulmonary toxicity in patients receiving bleomycin included: age > 40, pre-existing pulmonary disease, renal dysfunction (Cr > 1.5, EGFR < 80), smoking history, cumulative bleomycin dose (>300 units), the need for supplemental oxygen, mediastinal radiation pre- or post-treatment with bleomycin. If a variable did not exist (i.e. no patients were on supplemental oxygen), then it was not included in the multivariate analysis. In addition, given the small sample size, if there was a variable with missing data, it was omitted from the multivariate analysis. 

All statistical analyses were performed using SAS Version 9.4 (SAS Institute, Cary, NC). Hypothesis testing was two-sided and conducted at the 5% level of significance. Baseline and treatment characteristics were summarized as median (range) for continuous variables and number of subjects (percentage) for categorical variables. Group comparisons of continuous variables were made with the Wilcoxon rank sum test, while comparisons of categorical variables were made with the Chi-square or Fisher’s exact test, as appropriate. The Kaplan-Meier method was used to estimate the probability of bleomycin-induced toxicity within 3, 6 and 9 months in each treatment group by comparison between groups made with the Log-rank test. The Cox proportional hazards regression model was used to estimate crude and adjusted hazard ratios and corresponding 95% confidence intervals (CI) relating treatment with bleomycin toxicity. Both univariate and multivariate cox proportional hazards analyses were performed. The multivariable analyses included adjustment for baseline covariates: age at diagnosis, pulmonary disease and gender.

## Results

**FIGURE 1:**
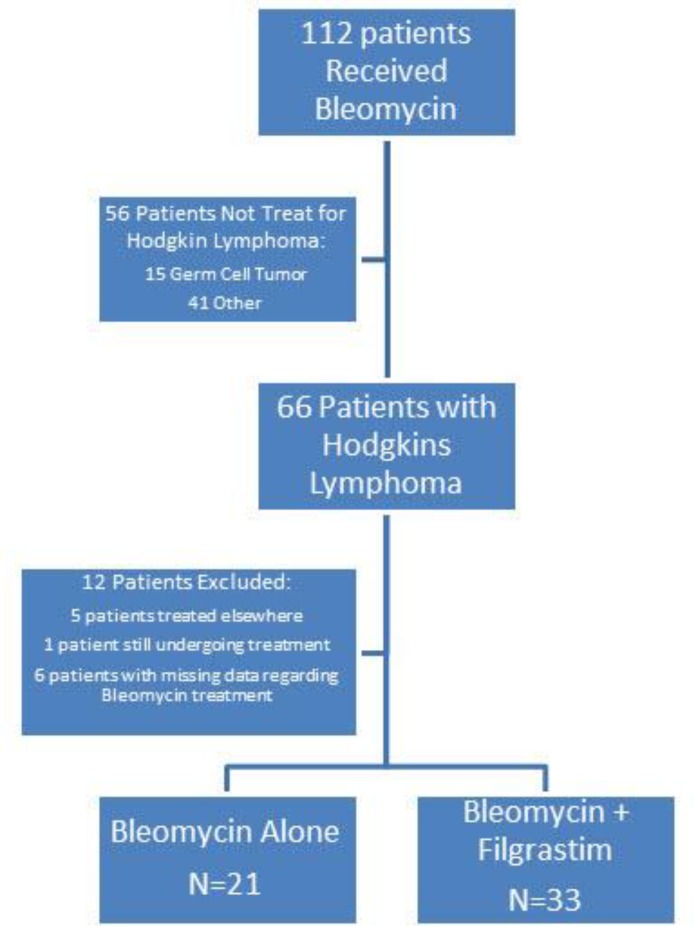
Study Outline

 A total of 112 patients who received at least one dose of bleomycin between January, 2003 and July, 2015 were identified from the Mount Sinai Data Warehouse. Sixty-six patients received bleomycin for a diagnosis other than Hodgkin lymphoma and were excluded from the study. Twelve patients who received bleomycin for a diagnosis of HL met exclusion criteria and were not included in the final analysis (Figure 1). 

Of the remaining 54 patients, 21 received bleomycin alone and 33 received bleomycin and G-CSF. Twenty-six patients were female and 28 were male. Nine patients (43%) in the bleomycin-only group and 13 patients (39%) in the bleomycin plus G-CSF group were over 40 years of age. There was no statistically significant difference between the demographics or risk factors in the two groups (Table 2). 

**Table 2:** Patient Characteristics

	**Bleomycin ** **Alone** **N=21**	**Bleomycin +** **Filgrastim** **N=33**	**P-value**	**Overall** **N=54**
**Gender** Female Male	9 (43%) 12 (57%)	17 (52%) 16 (48%)	0.5348	26 (48%) 28 (52%)
**Race** African American Caucasian Hispanic/Latino Other	9 (43%) 6 (29%) 1 (5%) 5 (24%)	4 Missing 17 (59%) 6 (21%) 0 (0%) 6 (21%)	0.5434	4 Missing 26 (52%) 12 (24%) 1 (2%) 11 (22%)
**Stage** 1 2 3 4	4 Missing 1 (6%) 5 (29%) 4 (24%) 7 (41%)	1 (3%) 13 (39%) 6 (18%) 13 (39%)	0.8149	4 Missing 2 (4%) 18 (36%) 10 (20%) 20 (40%)
**Renal Function** No Yes	2 Missing 18 (95%) 1 (5%)	33 (100%) 0 (0%)	0.3654	2 Missing 51 (98%) 1 (2%)
**Pulmonary Disease** No Yes	19 (90%) 2 (10%)	26 (78%) 7 (21%)	0.4559	45 (83%) 9 (17%)
**Age**	32 [19-67]	28 [9-78]	0.6129	31.5 [9-78]
**Age>40** No Yes	12 (57%) 9 (43%)	20 (61%) 13 (39%)	0.8007	32 (59%) 22 (41%)
**BMI**	23.8 [17.9-34.0]	24.6 [16.3-37.1]	0.9372	23.9 [16.3-37.1]
**Ever Smoked** No Yes	6 Missing 8 (53%) 7 (47%)	24 (73%) 9 (27%)	0.2063	6 Missing 32 (67%) 16 (33%)
**Total Bleo Dose>300** No Yes	21 (100%) 0 (0%)	33 (100%) 0 (0%)	NE	54 (100%) 0 (100%)
**Supplemental O2** No Yes	1 Missing 20 (100%) 0 (0%)	33 (100%) 0 (0%)	NE	1 Missing 53 (100%) 0 (0%)
**Mediastinal Radiation Pre-Chemo** No Yes	20 (100%) 0 (0%)	33 (100%) 0 (0%)	NE	53 (100%) 0 (0%)
**Mediastinal Radiation Post-Chemo** No Yes	1 Missing 16 (80%) 4 (20%)	27 (82%) 6 (18%)	1.000	1 Missing 43 (81%) 10 (19%)

NE: Not Estimable

The following risk factors were not present in the cohort and so these were not considered for multivariate analysis: cumulative bleomycin dose greater than 300 units, supplemental oxygen, or pre-chemotherapy radiation. 

Ten of the 54 patients who received bleomycin met the criteria for BIP. Of these 10 patients, 3 patients received bleomycin alone and 7 received a combination of bleomycin and G-CSF. The difference between these groups was not statistically significant (p = 0.50). Crude hazard ratio for development of pulmonary toxicity in the bleomycin and G-CSF cohort was 1.58 (95% CI: 0.41-6.12). On multivariate analysis, the hazard ratio for development of pulmonary toxicity was 1.71 (95% CI: 0.43-6.81).

Median follow-up was 24.9 months (range 2.5-122.4) for all patients, 29.5 months (range 3.3-122.4) for the bleomycin-only group, and 24.8 months (range 2.5-59.2) for the bleomycin and G-CSF group. There was no significant difference in the follow-up period between the two treatment groups (p=0.13). The median number of days to administration of G-CSF after the first dose of bleomycin was 14 (range 0-153). 

We performed subgroup analysis to determine the time to bleomycin toxicity after the start of treatment. In our cohort, all pulmonary toxicity occurred within the first 9 months after the start of treatment (Table 3). 

**Table 3:** Bleomycin Induced Lung Toxicity

	**Bleomycin ** **Alone** **N=21**	**Bleomycin +** **Filgrastim** **N=33**	**P-value**	**Overall** **N=54**	**Crude** **Hazard Ratio** **95% CI**	**Multivariable** c **Hazard Ratio** **95% CI**
Bleomycin Toxicity No Yes	18 (86%) 3 (14%)	26 (78%) 7 (22%)	0.5042	44 (81%) 10 (19%)	1.58 [0.41-6.12] P=0.5060	1.71 [0.43-6.81] P=0.4458
Probability of Bleomycin Toxicity Within 3 Months of Start of Treatment^a^	5% [1%-29%]^b^	9% [3%-26%]^b^		7% [3%-19%]^b^		
Probability of Bleomycin Toxicity Within 6 Months of Start of Treatment^a^	10% [3%-34%]^b^	22% [11%-41%]^b^		17% [9%-30%]^b^		
Probability of Bleomycin Toxicity Within 9 Months of Start of Treatment^a^	14% [5%-39%]^b^	22% [11%-41%]^b^		19% [11%- 33%]^b^		
Months of Follow-up Post Start of Treatment^a^ Median [ Min-Max]	29.5 [3.3-122.4]	24.8 [2.5-59.2]	0.1039	24.9 [2.5-122.4]		
Days from First Bleomycin Dose to Filgrastim administration Median [Min-Max]	NE	14 [0-153]	NE	2 [0-153]		

NE: Not Estimable;

a) Start of Treatment is Date of First Bleomycin Dose given if Bleomycin Alone or Date of First filgrastim administration or Date of First Bleomycin Dose given (whichever came last) if Bleomycin+filgrastim;

b) 95% confidence interval;

c) Adjusted for Age, Pulmonary Disease and Gender.

## Discussion

 Our study did not find any statistically significant increase in pulmonary toxicity in patients receiving bleomycin and G-CSF as compared to those patients receiving bleomycin alone. These findings are consistent with those derived from the most recent retrospective studies in the Mayo Clinic in which patients received bleomycin between 2000 and 2012^   7 ^. In an earlier retrospective study from the Mayo clinic, patients who developed BIP between 1986 and 2003 were found to have a worse 5-year overall survival compared to those who did not develop pulmonary toxicity^        5 ^. In this study, the investigators also identified risk factors for BIP and concluded that age greater than 40, first-line therapy with ABVD, and G-CSF were associated with BIP. Another more recent study also found G-CSF to be a risk factor for BIP, but 5-year overall survival was not affected those who developed BIP. It is unclear what factors may account for the discrepant results regarding the role of G-CSF and pulmonary toxicity found in these studies and our current study. Perhaps there were unmeasured variables that led to an increased risk for pulmonary toxicity as compared to the more recent cohort.

Another explanation is that underlying genetic polymorphisms exist to explain the risk for BIP as opposed or in addition to environmental or iatrogenic risk factors. This theory was demonstrated in a recently published study, revealing the presence of a single nuclear polymorphism (SNP) as a risk factor for BIP^   20 ^. However, this SNP was a risk factor for chronic pulmonary changes, but was not associated with any acute pulmonary toxicity. Our population had a significant number of African Americans and it is possible that they do not carry a SNP that would put them at increased risk for BIP, thus resulting in a negative study. While SNPs may contribute to the complex understanding of who will develop BIP, it does not seem to be the whole story. Another explanation for these conflicting results is simply a limitation of retrospective studies. 

Our study was not designed to evaluate other risk factors that could contribute to pulmonary toxicity in this patient population. We collected data on 7 potential risk factors that have been documented in previous studies in order to control for possible confounding variables between our two cohorts^5^^,^^17^^-^^19^. However, 3 of the risk factors (cumulative bleomycin dose greater than 300 units, supplemental oxygen, or pre-chemotherapy radiation) were not identified in our patient population. In addition, given the small size of the study, we were unable to control for any variables that had missing data as it would further exclude patients. However, on multivariate analysis, we did control for age, previous pulmonary disease and gender. Age and previous pulmonary disease have consistently shown to be risk factors for BIP, so controlling for these variables was important for the integrity of our results.

Interestingly, the timing of pulmonary toxicity varied between our two cohorts. In the bleomycin and G-CSF cohort, all 7 patients who developed pulmonary toxicity did so within the first six months of starting therapy (i.e. during the treatment period), with the majority developing toxicity from 3-6 months of starting therapy. While the number of patients developing toxicity in the bleomycin-only cohort was small, patients developed toxicity more gradually with toxicity occurring up to 6-9 months from start of therapy. While the authors could not make definitive conclusions regarding this finding, it may suggest that in those predisposed to developing bleomycin-induced pulmonary toxicity, G-CSF will accelerate the development of this toxicity (Table 3). 

One of the limitations of the study was the small sample size. Although we reviewed the records for all patients treated for HL at our institution during the specified time period, the sample size was small and any definitive conclusions are difficult given the wide confidence intervals. However, this study contributes to the existing literature questioning the increased risk for BIP when G-CSF is used in HL patients receiving bleomycin. 

As this field evolves, this question may be less pertinent. The RATHL study examining management of patients with PET/CT negative disease after 2 cycles of ABVD may minimize exposure to prolonged combination chemotherapy for a large subset of HL patients^   21 ^. In addition, the Millennium Study may reveal that a brentuximab-vedotin containing regimen is superior to bleomycin changing the management paradigm of this disease. However, currently, ABVD remains the standard of care and strategies to minimize treatment complications should be developed. Given that the Millennium study allowed for the use of G-CSF, if clinically indicated, results from a secondary analysis in this study may shed additional light on whether the use of G-CSF contributes to BIP. If this analysis is unable to be completed, then there is equipoise for a future prospective study randomizing patients to bleomycin alone compared to bleomycin and G-CSF in order to further clarify this unanswered question. 

NCCN guidelines recommend growth factor support when the risk of febrile neutropenia is >20% and recommends consideration for G-CSF use when the risk is <20%. ABVD is categorized in the intermediate risk group with a 10-20% risk of febrile neutropenia^   22 ^. We feel that growth factor use should not be routinely administered during the first cycle of ABVD. However, for patients who do not meet white blood cell count parameters on the day of subsequent planned treatment or just prior to subsequent treatment, we recommend administering one dose of G-CSF at 5 mcg/kg in order to maintain dose intensity of chemotherapy for this patient population. In addition, if a patient develops febrile neutropenia, we recommend the use of G-CSF with subsequent cycles to minimize the duration of neutropenia. Pegfilgastrim use should be avoided given the short duration between chemotherapy doses.
